# Dexmedetomidine Improves Lung Function by Promoting Inflammation Resolution in Patients Undergoing Totally Thoracoscopic Cardiac Surgery

**DOI:** 10.1155/2020/8638301

**Published:** 2020-09-08

**Authors:** Junji Cui, Mintai Gao, Hongqian Huang, Xiaoyan Huang, Qingshi Zeng

**Affiliations:** ^1^Department of Anesthesiology, Guangdong Cardiovascular Institute, Guangdong Provincial People's Hospital, Guangdong Academy of Medical Sciences, Guangzhou 510080, China; ^2^Department of Anesthesiology, Guangdong Provincial People's Hospital Zhuhai Hospital (Zhuhai Golden Bay Center Hospital), Zhuhai 519040, China; ^3^Department of Clinical Laboratory, Guangdong Provincial People's Hospital Zhuhai Hospital (Zhuhai Golden Bay Center Hospital), Zhuhai 519040, China

## Abstract

**Objective:**

Totally thoracoscopic cardiac surgery under cardiopulmonary bypass combined with one-lung ventilation has been identified as the trend in cardiac surgery. The aim of this study was to examine the effects of the selective *α*_2_ adrenergic receptor agonist dexmedetomidine on the pulmonary function of patients who underwent mitral valve surgery using the totally thoracoscopic technique.

**Methods:**

Fifty-seven patients who underwent thoracoscopic mitral valve surgery between July 2019 and December 2019 were selected. The patients were randomly divided into the control (Con) group (*n* = 28) and the dexmedetomidine (DEX) group (*n* = 29) using the random number table method. Arterial blood gas analyses were performed, and the oxygenation (PaO_2_/FiO_2_) and respiratory indexes (P(A-a)O/PaO_2_) were calculated 5 min after tracheal intubation (T1), 2 h after operation (T2), 6 h after operation (T3), and 24 h after operation (T4). Moreover, the serum cytokines interleukin-6 (IL-6), tumor necrosis factor-*α* (TNF-*α*), and intercellular adhesion molecule-1 (ICAM-1) were detected using the enzyme-linked immunosorbent method at all time points. Chest radiography was performed 24 h after surgery. Peripheral blood samples were collected before and after the operation for a complete hemogram. Additionally, the procalcitonin concentration was measured and recorded when the patients were transported to the intensive care unit (ICU). The postoperative extubation time, length of ICU stay, and pulmonary infection rate were also recorded.

**Results:**

Inflammatory reaction after surgery was evident. However, the inflammatory cytokines IL-6, TNF-*α*, and ICAM-1 in the DEX group were lower than those in the Con group after surgery (T2 to T4; *P* < 0.05). Neutrophil counts and procalcitonin concentration were higher in the Con group than in the DEX group (*P* < 0.05). In addition, in the DEX group, pulmonary exudation on chest radiography was lower, and pulmonary function, as shown by an increase in oxidation index and decrease in the respiratory index, improved after surgery (*P* < 0.05). Moreover, the duration of mechanical ventilation in the Con group was 3.4 h longer than that in the DEX group.

**Conclusion:**

Dexmedetomidine has a protective effect on pulmonary function in patients undergoing mitral valve surgery using a totally video-assisted thoracoscopic technique, which may be related to a reduction in the concentration of inflammatory cytokines in the early perioperative period.

## 1. Introduction

Totally thoracoscopic cardiac surgery has developed rapidly in clinical practice due to its minimally invasive technique and rapid recovery [1]. Thoracoscopic cardiac surgery requires cardiopulmonary bypass (CPB) and the one-lung ventilation (OLV) technique. Both CPB and OLV can result in acute lung injury associated with the systemic inflammatory response syndrome (SIRS) and pulmonary ischemia-reperfusion injury, which can lead to pulmonary dysfunction and seriously affect a patient's prognosis [2–4]. SIRS caused by CPB has been one of the main factors that lead to postoperative lung injury after CPB. Activation of the complement system is considered the initiating factor of postoperative lung injury from CPB [5]. Both the classic and alternative pathways of the complement system are activated during the CPB process, with the intensity of the inflammatory response enhanced through interaction with inflammatory factors such as tumor necrosis factor-*α* (TNF-*α*), procalcitonin (PCT), interleukin-1 (IL-1), IL-2, IL-6, and IL-8. Neutrophils adhere to pulmonary vascular endothelial cells under the influence of inflammatory factors such as IL-1, IL-6, IL-8, and TNF-*α*; platelet-activating factors; and leukotriene B, which causes an increase in pulmonary capillary permeability and edema. The interaction and cascade of cytokines during OLV are the primary mechanisms that cause lung injury [6].

Dexmedetomidine (DEX) is a highly selective *α*_2_ adrenergic receptor (*α*2AR) that is widely used in perioperative cardiovascular surgeries. Specifically, DEX activates *α*_2_ adrenergic receptors and acts as a sedative agent by blocking signal transduction in the nucleus coeruleus near the fourth ventricle [7]. In recent years, clinical and basic research has confirmed that DEX has a protective effect against lung injury [8]. One study has shown that DEX can reduce lung inflammation in septic rats by inhibiting the TLR4/NF-*κ*B pathway [9]. DEX also increases the expression level of heme oxygenase-1 and the activity of superoxide dismutase in the lung during OLV, reducing oxidative stress and inflammation caused by OLV in patients with lung cancer during surgery, thereby improving intrapulmonary shunting and hypoxemia [10, 11]. In addition, DEX may reduce IL-6 levels and high-mobility group box-1 generation by inhibiting the activation of NF-*κ*B, which leads to a suppression of SIRS after CPB [12].

This study is aimed at examining the effects of DEX on inflammatory factors and respiratory indicators during thoracoscopic mitral valve surgery to provide clinical evidence for organ protection during cardiac surgery.

## 2. Materials and Methods

### 2.1. Study Design

The experimental methods of this study were designed in accordance with the Declaration of Helsinki. The clinical trial was registered under No. ChiCTR2000032652 in the Chinese Clinical Trial Registry, and patients provided informed consent after the study was approved by the Institute of Medical Ethics Committee at Guangdong Provincial People's Hospital (No. GDREC2020001H).

### 2.2. Study Patients and Protocol

The present study was conducted at Guangdong Provincial People's Hospital. Fifty-seven consecutive patients who underwent totally thoracoscopic valvular cardiac surgery between July 2019 and September 2019 were included after they were assessed for the following criteria: (1) patients aged between 18 and 65 years, (2) American Society of Anesthesiology score II or III and New York Heart Association class II or III, (3) an estimated extracorporeal circulation of ≤180 min, (4) no active infective endocarditis, and (5) a rheumatic factor (RF) level of <20 IU/mL.

The following patients were excluded: (1) elderly patients ≥ 70 years old; (2) patients without sick sinus syndrome and atrioventricular block (bradycardia, heart rate < 60 beats per minute, atrioventricular block above the first degree, or indoor conduction block); (3) patients accompanied with coronary heart disease or cerebrocardiac syndrome; (4) patients with Gold grade of >level 2; (5) patients with severe pulmonary hypertension, with a pulmonary artery systolic pressure of >60 mmHg; (6) patients with immune system diseases; (7) patients with abnormal renal function with a blood creatinine level of >110 *μ*mol/L; and (8) patients in whom CPB or the prevention of all-cause mortality from reoperation was difficult.

**Figure 1:**
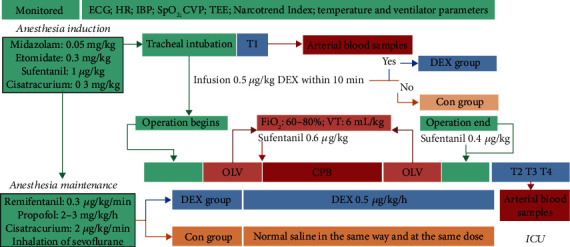
Flow diagram of the study protocol. (1) Dexmedetomidine (DEX) group. The patients in the DEX group received an infusion of 0.5 *μ*g/kg DEX within 10 min via a microinfusion pump after successful tracheal intubation. DEX at a dose of 0.5 *μ*g/kg/h was then continuously infused until the operation was completed. (2) The control (Con) group. The patients in the Con group were administered normal saline in the same way and at the same dose. Arterial blood samples (3 mL) were collected in the Con and DEX groups at 5 min (T1) after tracheal intubation and 2 h (T2), 6 h (T3), and 24 h (T4) after surgery. ECG: electrocardiography; HR: heart rate; HR: heart rate; CPB: cardiopulmonary bypass; OLV: one-lung ventilation; IBP: invasive blood pressure; VT: tidal volume; TEE: transesophageal echocardiography.

Patients were randomly divided into 2 groups according to the random number table method (Figure 1). In the DEX group, after successful tracheal intubation, the patients received an infusion of 0.5 *μ*g/kg DEX within 10 min using a microinfusion pump. DEX was then continuously infused at 0.5 *μ*g/kg/h until the operation was completed. In the control (Con) group, the patients were administered normal saline instead of DEX, but at the same rate and dose as in the experimental group.

### 2.3. Sample Size

Based on the main outcome measures (various points of pulmonary function) in our preliminary experiment, to achieve acceptable results, the sample allocation ratio of the two groups was 1.0, the test effect (1 − *β*) of the study was 0.9, and the significance level was *α* = 0.05. The PASS 11.0 software was used for calculating the sample size and the T2 time point oxygenation index (Con group 322 ± 45 and DEX group 370 ± 60). The maximum of the minimum sample size was 27 patients. Based on a potential dropout rate of 20%, each group needed 33 patients, and 72 patients were included in the actual study.

### 2.4. Anesthesia and Surgical Procedure

The patients, monitored using electrocardiography, underwent radial artery puncture and catheterization after entering the operating room. Intravenous injections of midazolam 0.05 mg/kg (Jiangsu Nhwa Pharmaceutical Co., Ltd., Jiangsu, China), sufentanil 1 *μ*g/kg (Yichang Humanwell Pharmaceutical Co., Ltd., Yichang, China), etomidate 0.3 mg/kg (Jiangsu Nhwa Pharmaceutical Co., Ltd., Jiangsu, China), and cisatracurium 0.3 mg/kg (Hengrui Medicine, Jiangsu, China) were used for the induction of anesthesia. Tracheal intubation was performed 5 min after induction using the Shiley Endobronchial Tube (Covidien, Medtronic, Inc., USA), and tracheal catheterization was performed using bronchoscopy. Next, a superior vena cava drainage tube and central venous catheters (B Braun, Ltd., Germany) were inserted through the right internal jugular vein.

During the maintenance of anesthesia, remifentanil (Yichang Humanwell Pharmaceutical Co., Ltd., Yichang, China) was infused with a micropump at a rate of 0.3 *μ*g/kg/min, propofol (Jiangsu Nhwa Pharmaceutical Co., Ltd., Jiangsu, China) at a rate of 2–3 mg/kg/h, and cisatracurium (Hengrui Medicine, Jiangsu, China) at a rate of 2 *μ*g/kg/min, with intermittent inhalation of sevoflurane (Hengrui Medicine). The Narcotrend Index was kept at 25–35, and the protective pulmonary ventilation strategy was adopted during OLV with the anesthesia system Aisys CS2 (GE Medical System Trade Development-Shanghai Co., Ltd.). The fraction of inspiration O_2_ (FiO_2_) was 50%–80%, the tidal volume was controlled at approximately 6 mL/kg, the respiratory rate was 16-22 times per minute, an inspiratory-to-expiratory ratio of 1 : 2 was performed, the transcutaneous oxygen saturation (SpO_2_) was maintained at >90%, and an end-expiratory pressure of 3–5 cmH_2_O (0.29–0.49 kPa) was used.

After anesthesia induction, for achieving a good operation position, the CPB was established through the superior vena cava and femoral vein-to-artery cannulation. A right anterior external fourth thoracotomy of approximately 3.5 cm was performed. At the level of the axillary midline, an endoscope was inserted through the fifth intercostal space, inserting Chitwood aortic occlusive forceps through the fourth intercostal and parallel atrial sulcus incision of the left atrium. During the operation, the CPB was operated with Stockert S5 (Solin Medical-Shanghai, Co., Ltd.), an artificial cardiopulmonary system, and a CPB perfusion flow of 1.8–2.4 L/m^2^/min was maintained. The intraoperative hematocrit level was controlled at 25%–30%, and the internal environment was stable. Vacuum-assisted venous drainage technology (negative pressure controlled at −50 to −35 mmHg, 1 mmHg = 0.133 kPa) with whole-body cooling (moderate degrees of systemic hypothermia at 28°C) was used intraoperatively. After the completion of the intracardiac operation, rewarming to a rectal temperature of 36°C was initiated. Once the results of the qualitative and quantitative analyses of cardiac structure and function using the esophageal echocardiography technique were found to be satisfactory, CPB was stopped. Before chest closure, sputum aspiration and atelectasis were performed. In both patient groups, the basic balance between liquid intake and output was maintained during operation.

### 2.5. Inflammatory Cytokine Detection and Outcome Parameter Measurements

In both groups, arterial blood samples (3 mL) were collected at all time points (T1–T4). RapidPoint 500 (R) (Siemens Healthcare Diagnostics) blood gas analysis system was used to calculate the oxidation index (OI) and respiratory index (RI). The remaining arterial blood was stored in a tube containing coagulant ethylenediaminetetraacetic acid (Zhejiang Huafu Medical Equipment Co., Ltd., China), allowed to stand for 1 h, and then centrifuged at room temperature at 3500 r/min in a BY-600A centrifuge (Bai yang Medical, Guangdong, China) for 10 min. The supernatant was then collected for the detection of TNF-*α*, IL-6, and intercellular adhesion molecule-1 (ICAM-1).

Peripheral venous blood samples were collected before and after the operation for a complete hemogram (white blood cell (WBC) counts, neutrophil counts, hemoglobin (Hb) levels, and platelet counts) and for assessing the levels of creatinine, blood urea nitrogen, alanine aminotransferase (ALT), aspartate aminotransferase (AST), activated partial thromboplastin time (APTT), and prothrombin time (PT). In addition, procalcitonin (PCT) activity was measured using the ELISA method in accordance with the manufacturer's instructions (KeyGen Biotech. Co., Ltd., Nanjing, China).

In addition, the postoperative extubation time, length of intensive care unit (ICU) stay, and the incidence of pulmonary infections were recorded.

### 2.6. Statistical Analyses

The SPSS 25.0 statistical software was used for the data analysis. Numerical data are described as numbers or rates. The Pearson *χ*^2^ test was used for intergroup comparisons. The measurement data are described as mean ± standard deviation. The *t*-test was used for intergroup comparisons. Repeated-measures data were analyzed using repeated-measures analysis of variance. Differences were considered significant when the two-tailed *P* values were <0.05.

## 3. Results

### 3.1. No Significant Difference in Basic Patient Characteristics Was Found between the Con and DEX Groups

**Figure 2:**
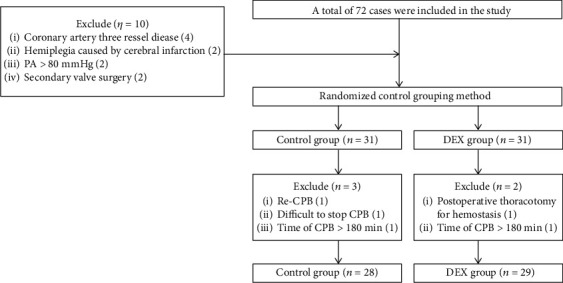
Flow chart of patient selection. In total, 76 patients were enrolled, of whom 16 dropped out of the study after randomization (shorter CPB than planned in 6 patients, surgical complications of thoracotomy and secondary chest closure in 5, irregular randomization in 1, heavily calcified large blood vessels that made cannulation impossible for CPB in 1, and loss of follow-up in 3).

In accordance with the inclusion and exclusion criteria, 57 patients were included in the present study (Figure 2). As shown in Table 1, no statistically significant differences were found between the Con and DEX groups in terms of sex, age, body mass index, cardiac function grade, left ventricular ejection fraction, pulmonary artery systolic pressure, aortic occlusion time, extracorporeal circulation time, surgical type, or comorbidities such as diabetes, grade 3 hypertension, and atrial fibrillation (*P* > 0.05). As shown in Table 2, there were no significant differences found between the two groups in terms of preoperative and postoperative hemoglobin levels, liver function, coagulation parameters, or renal function (*P* > 0.05). The mean postoperative creatinine level in the Con group was 11 *μ*mol/L higher than that in the DEX group, but this did not affect normal renal function. As shown in Table 3, no significant difference in postoperative 24 h left ventricular ejection fraction was found between the two groups (*P* > 0.05). All patients in both groups successfully underwent the operation and were discharged after recovery.

### 3.2. DEX Effectively Reduced the Inflammatory Response after Thoracoscopic Cardiac Surgery

**Figure 3:**
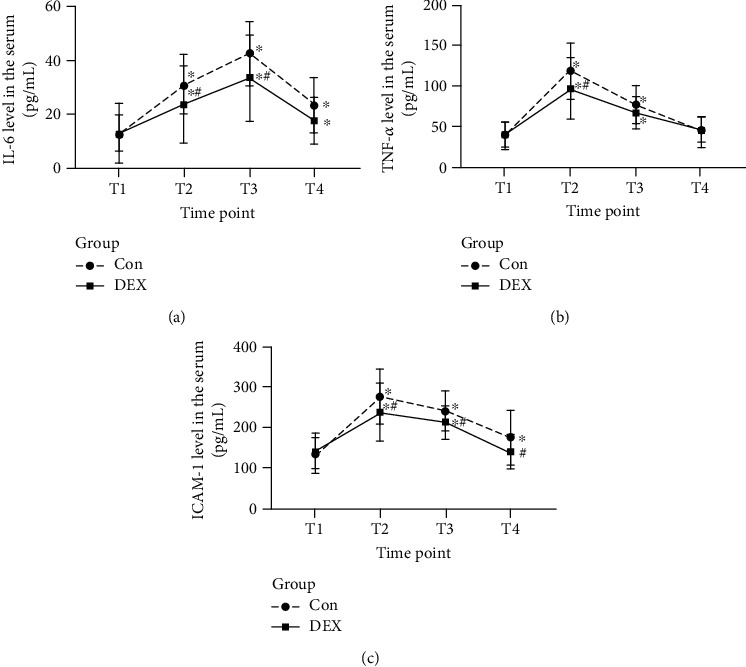
Changes in the perioperative serum levels of inflammatory factors. (a) Serum IL-6 level. (b) Serum TNF-*α* level. (c) Serum ICAM-1 level. ^∗^A higher level with a *P* value of <0.05 for a within-group comparison at the time point T1. ^#^A higher level with a *P* value of <0.05 in comparison with the Con group at the same time point.

As shown in Figure 3, in the Con group, the serum IL-6 and ICAM-1 levels after thoracoscopic cardiac surgery were significantly higher at T2–T4 (*P* < 0.05 vs. T1; Figures 3(a) and 3(c)). The serum TNF-*α* level was also significantly higher at T2–T3 than at T1 (*P* < 0.05 vs. T1) but gradually decreased by T4 (Figure 3(b)). In the DEX group, the serum IL-6, ICAM-1, and TNF-*α* levels were higher at T2–T3 (*P* < 0.05 vs. T1), but these levels were significantly lower at T4 than at T1 (*P* > 0.05 vs. T1; Figures 3(a)–3(c)). The serum IL-6 and ICAM-1 levels in the DEX group were significantly lower at T2, T3, and T4 (*P* < 0.05 vs. the Con group; Figures 3(a) and 3(c)). In addition, the decrease in the serum TNF-*α* level at T2 was significantly greater in the DEX group (*P* < 0.05 vs. Con group; Figure 3(b)).

### 3.3. DEX Reduced the Serum PCT Concentration and Neutrophil Count in Peripheral Blood

As shown in Table 2, the postoperative complete blood cell count (white blood cell and neutrophil counts) was lower in the DEX group than in the Con group (*P* < 0.05). Moreover, the serum PCT concentration was lower in the DEX group than in the Con group (*P* < 0.05), suggesting that the risks of inflammation and infection were lower in the DEX group.

### 3.4. DEX Attenuated Postoperative Pulmonary Exudation after Thoracoscopic Cardiac Surgery

**Figure 4:**
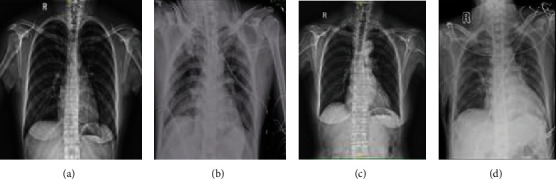
Digital radiography. Representative digital radiograph of a patient in the Con group taken (a) before surgery and (b) 1 d after surgery. Representative digital radiograph of a patient in the DEX group taken (c) before surgery and (d) 1 d after surgery.

As shown in the digital radiography image in Figure 4, the two groups showed clear lung texture before surgery with no obvious parenchymal lesions and normal brightness of the lung field. Postoperative changes in lung texture were observed in both groups; however, lung exudation was more obvious in the Con group than in the DEX group. These results show that DEX could alleviate postoperative pulmonary exudation after thoracoscopic cardiac surgery.

### 3.5. DEX Improved Pulmonary Dispersion, Ventilation, and Oxygenation after Thoracoscopic Cardiac Surgery

The RI reflects lung diffusion and ventilation function, while the oxygenation index (OI) reflects pulmonary oxygenation function.

**Figure 5:**
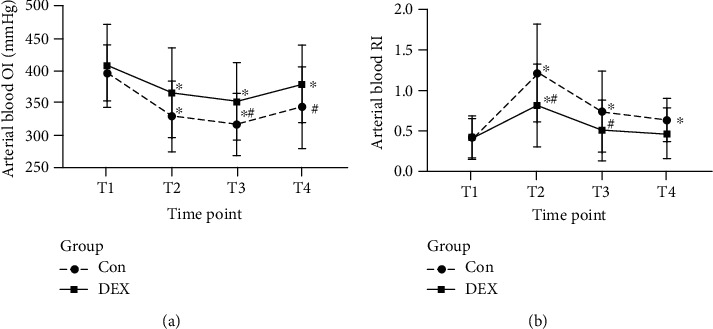
Changes in perioperative pulmonary function in both groups. (a) Arterial oxygenation index. (b) Respiratory index based on the assessment of arterial blood. ^∗^A higher level with a *P* value of <0.05 for a within-group comparison at the time point T1. ^#^A higher level with a *P* value of <0.05 in comparison with the Con group at the same time point.

After thoracoscopic cardiac surgery, the OI of the patients in the Con group was lower at T2–T4 (*P* < 0.05 vs. T1; Figure 5(a)), reaching its lowest value at T3. The OI at T3 showed a 21% decrease compared to that at baseline. In addition, the RI was higher at T2–T3 (*P* < 0.05 vs. T1; Figure 5(b)), reaching its highest level at T2, when the RI was three times the baseline value.

In the DEX group, the OI was lower at T2–T3 than at T1 (*P* < 0.05 vs. T1; Figure 5(a)) and reached its lowest value at T3, but the OI decreased by only 15% compared to the baseline value. The RI was also higher at T2–T3 (*P* < 0.05 vs. T1) and showed the highest value at T2; however, at T2, the RI was only 1.95-times the baseline value (Figure 5(b)). The OI levels at T2, T3, and T4 in the DEX group were significantly higher than those in the Con group (*P* < 0.05 vs. the Con group; Figure 5(a)). In addition, the RI levels at T2 were significantly lower in the DEX group (*P* < 0.05 vs. the Con group; Figure 5(b)).

### 3.6. DEX Reduced the Mechanical Ventilation Time in the ICU

As shown in Table 4, the extubation time in the Con group was 3.4 h longer than that in the DEX group (*P* < 0.05). However, no significant differences were found between the groups in terms of postoperative ICU stay and pulmonary infection incidence.

## 4. Discussion

This large-sample retrospective study demonstrated that administering DEX to manage perioperative anesthesia can reduce the impact of CPB, surgery, and anesthesia on various organs of the body, reducing the occurrence of postoperative complications and improving prognosis [13]. While DEX has a high potential of benefit in this context, no uniform dose standard has been established for DEX in cardiovascular surgery. To avoid transient hypertension and excessive inhibition of the cardiovascular system, 0.5 *μ*g/kg [14] administered over <10 min was selected as the loading dose, followed by a continuous infusion rate of 0.5 *μ*g/kg/h until the end of surgery.

With the development of new technology in cardiac surgery, the associated mortality rate has noticeably decreased. However, postoperative pulmonary insufficiency remains as one of the main causes of postoperative mortality [15]. The systemic inflammatory response associated with CPB, such as the activation of the complement system; cytokines TNF-*α*, IL-6, and other inflammatory mediators; and the aggregation and activation of neutrophils in the lung, is involved in the process of lung injury. In addition, lung ischemia-reperfusion injury, which includes ischemia and cell damage caused by reperfusion and subsequent oxygen free radical release, various inflammatory mediators, and factor secretion, eventually leads to lung dysfunction, which is characterized by an increase in pulmonary vascular permeability, pulmonary edema, elevated pulmonary vascular resistance, and leukocyte infiltration [16]. In addition, OLV is accompanied by a large increase in plasma levels of inflammatory factors, resulting in systemic and pulmonary inflammatory responses, which may lead to a relatively high tidal volume and airway pressure and to overexpansion and collapse of the alveoli [17, 18]. Compared with the traditional techniques, totally thoracoscopic cardiac surgery requires more time for CPB and OLV [19], which may lead to an increase in the risk of lung injury. Moreover, owing to pulmonary ischemia-reperfusion and swelling mechanical injury, 8.0% of patients undergoing totally thoracoscopic cardiac surgery develop unilateral pulmonary edema after the operation and have a higher risk of death [20, 21]. Therefore, lung protection is of great importance to the prognosis of totally thoracoscopic cardiac surgery.

A number of inflammatory factors are involved in the damage to the lungs and other important organs after CPB [22, 23]. Evidence shows that neutrophils are closely related to the degree of lung injury and the levels of inflammatory factors [24]. IL-6 has proinflammatory effects on various cells during the acute inflammatory response. It can induce multiple-organ involvement, mainly including the respiratory system and central nervous systems [25]. TNF-*α* is considered an early inflammatory responder and can increase the permeability of microcirculatory vessels and affect the RI of patients after CPB and is closely related to multiple-organ dysfunction after CPB [26]. Plasma ICAM-1 is considered a key molecule in mediating the inflammatory process and plays an important role in all phases of an inflammatory reaction. In the inflammatory microenvironment, ICAM-1 can be activated in various cells. ICAM-1 binds to lymphocyte function-related antigen 1, the macrophage-1 molecule, and CD43 on the leukocyte surface, promoting the adhesion of the leukocyte to vascular endothelial cells, with an increased expression as the degree of lung injury increases [27]. *In vivo* and *in vitro* studies have revealed that CPB causes the cascade release of TNF-*α*, IL-6, IL-8, IL-10, and other proinflammatory and anti-inflammatory mediators and promotes the generation of PCT, which more sensitively reflects the severity of the inflammatory response in the early postoperative stage [28]. The risk of various postoperative complications such as cardiopulmonary insufficiency and SIRS increases in patients after coronary bypass or valve replacement cardiac surgery when the PCT concentration exceeds the threshold of 2 ng/mL after surgery [29]. Therefore, IL-6, TNF-*α*, and ICAM-1 were used as inflammatory indicators in this study. PCT can reflect the response state of intraoperative inflammation in patients and is a good indicator of postoperative infections. After thoracoscopic cardiac surgery, when the patient is still under anesthesia, a blood gas analysis can objectively reflect the patient's ventilation and ventilatory function, as it is not affected by the environment and patient's cooperation. The RI refers to the ratio of the difference in alveolar-arterial oxygen tension to the partial pressure of arterial oxygen, which means that an increase in RI indicates a decrease in lung dispersion and ventilation. A decrease in OI, which indicates pulmonary ventilatory function, indicates a decrease in pulmonary oxygenation function [30]. In the present study, the levels of IL-6, TNF-*α*, and ICAM-1 significantly increased at T2 and T3, accompanied by higher RI and lower OI than those before the operation, indicating that the inflammation after thoracoscopic cardiac surgery is noticeably enhanced and pulmonary function was impaired.

Preventing and reducing inflammation during thoracoscopic heart surgery are important methods for preserving lung function. The development of strategies to optimize the anesthesia scheme so as to reduce perioperative traumatic stress experienced by patients, reduce the production of inflammatory mediators, further improve the pulmonary function of patients, and promote the rapid recovery of patients is an important topic for anesthesiologists. DEX selectively activates the *α*_2_ adrenaline receptors to reduce sympathetic tone and indirectly improves vagal tone, thereby activating the cholinergic anti-inflammatory pathway and reducing the systemic inflammatory response [31]. In addition, DEX can inhibit the production of inflammatory factors such as TNF-*α* and IL-6 during CPB [32], alleviate the infiltration of inflammatory cells into the alveolar cavity, and reduce the levels of TNF-*α* and IL-1 levels in plasma and alveolar fluid by inhibiting the activation of NF-*κ*B, finally reducing the inflammatory response of rat lung tissues and playing a role in lung protection in rats with lung injury [33]. In the present study, we found that neutrophil counts and the levels of IL-6, TNF-*α*, and ICAM-1 after surgery were lower in the DEX group than in the Con group. The PCT concentration was higher in the Con group than in the DEX group. Pulmonary exudation was reduced in the DEX group based on the basis of radiography, and pulmonary function had improved in the DEX group, as evidenced by the increase in the OI and decrease in the RI at T2 to T4 as compared with T1. Moreover, the duration of mechanical ventilation in the Con group was 3.4 h longer than that in the DEX. These results suggest that DEX can reduce postoperative exudation and protect lung function, which may be related to a reduction in inflammatory factors. However, no significant difference was found between the groups in terms of postoperative ICU time and pulmonary infection rate. Therefore, whether increasing the dose of DEX or whether extending the duration of its action affects postoperative ICU time and pulmonary infection rates must be further verified.

In summary, the clinical use of dexmedetomidine is safe and feasible and has a certain value in improving pulmonary function after totally video-assisted thoracoscopic mitral valve surgery under CPB combined with OLV, further improving the effect of minimally invasive cardiac surgery.

## Figures and Tables

**Table 1:** Demographic characteristics of participants in the two groups.

	Con group (*n* = 28)	DEX group (*n* = 29)	*P* value
Age (y)	49.89 ± 13.89	48.04 ± 14.40	0.625
Sex (male/female)	16/12	18/11	0.705
Body mass index (kg/m^2^)	22.10 ± 3.02	22.40 ± 3.40	0.722
NYHA classification, *n* (%)			0.669
II	26 (92.9)	26 (89.7)	
III	2 (7.7)	3 (10.3)	
LVEF (%)	63.89 ± 6.31	62.28 ± 4.97	0.286
PASP (mmHg)	39.39 ± 14.11	34.31 ± 11.96	0.148
Duration of CPB (min)	135.86 ± 33.75	134.71 ± 25.97	0.888
Aortic cross-clamping (min)	92.46 ± 27.53	85.32 ± 19.14	0.265
Operation type, *n* (%)			0.760
MVR	4 (14.3)	5 (82.8)	
MVP	24 (85.7)	24 (17.2)	
Comorbidities, *n* (%)			
Grade 3 hypertension	2 (7.7)	3 (10.3)	0.669
Diabetes mellitus	2 (7.1)	1 (3.4)	0.532
Atrial fibrillation	7 (25.0)	5 (17.2)	0.473

Quantitative data are presented as means ± standard deviations or numbers (percentages). NYHA: New York Heart Association; PASP: pulmonary artery systolic pressure; LVEF: left ventricular ejection fraction; MVR: mitral valve replacement; MVP: mitral valvuloplasty.

**Table 2:** Perioperative blood biochemistry results of the patients in the two groups.

	Con group (*n* = 28)	DEX group (*n* = 29)	*P* value
Hemoglobin (g/L)			
Preoperative	12.48 ± 1.42	12.62 ± 1.53	0.725
Postoperative	11.11 ± 1.47	10.81 ± 1.38	0.425
Aspartate aminotransferase (U/L)			
Preoperative	27.44 ± 9.17	25.57 ± 7.09	0.392
Postoperative	88.43 ± 21.86	82.19 ± 23.21	0.302
Alanine aminotransferase (U/L)			
Preoperative	22.68 ± 11.36	20.50 ± 10.14	0.266
Postoperative	34.01 ± 12.70	30.39 ± 11.57	0.392
Urea nitrogen (mmol/L)			
Preoperative	5.54 ± 1.71	5.38 ± 1.29	0.623
Postoperative	7.57 ± 2.63	7.15 ± 2.45	0.536
Creatinine (*μ*mol/L)			
Preoperative	71.36 ± 20.68	71.03 ± 18.92	0.951
Postoperative	92.07 ± 25.37	81.66 ± 19.75	0.089
Platelet (10^9^/L)			
Preoperative	189.46 ± 36.40	194.38 ± 38.39	0.622
Postoperative	153.25 ± 43.10	151.17 ± 41.58	0.854
APTT (sec)			
Preoperative	31.36 ± 3.96	31.83 ± 3.94	0.659
Postoperative	42.49 ± 12.07	42.98 ± 6.99	0.852
PT (sec)			
Preoperative	14.89 ± 4.99	15.43 ± 4.96	0.679
Postoperative	14.29 ± 2.68	13.70 ± 2.32	0.376
WBC (10^9^/L)			
Preoperative	5.45 ± 1.12	5.38 ± 0.94	0.789
Postoperative	16.69 ± 5.71	13.93 ± 3.50	0.031
Neutrophil (10^9^/L)			
Preoperative	3.64 ± 0.79	3.48 ± 1.03	0.506
Postoperative	14.26 ± 5.31	11.46 ± 3.42	0.021
Procalcitonin (ng/mL)			
Postoperative	7.55 ± 6.82	3.14 ± 2.27	0.020

Quantitative data are presented as means ± standard deviations. WBC: white blood cell; APTT: activated partial thromboplastin time; PT: prothrombin time.

**Table 3:** Postoperative vasoactive-inotropic score (VIS) and left ventricular ejection fraction (LVEF) of the patients in the two groups.

	Con group (*n* = 28)	DEX group (*n* = 29)	*P* value
VIS (*μ*g/kg/min)			
Postoperative 2 h	6.18 ± 3.57	4.88 ± .3.28	0.158
Postoperative 6 h	5.20 ± 3.24	3.34 ± 1.20	0.006
LVEF (%)			
Postoperative 24 h	58.18 ± 5.81	57.00 ± 5.10	0.419

Quantitative data are presented as means ± standard deviations. VIS: vasoactive‐inotropic drug score = dopamine (*μ*g/kg/min)∗1 + dobutamine (*μ*g/kg/min)∗1 + milrinone (*μ*g/kg/min)∗10 + adrenaline (*μ*g/kg/min)∗100 + norepinephrine (*μ*g/kg/min)∗100 + pituitrin (units/kg/min)∗1000; h: hour.

**Table 4:** Postoperative duration of extubation and ICU stay in the two groups.

	Con group (*n* = 28)	DEX group (*n* = 29)	*P* value
Duration of extubation (h)	8.782 ± 7.18	5.310 ± 4.37	0.031
Duration of ICU stay (h)	59.89 ± 37.74	49.41 ± 24.01	0.220
Pulmonary infection rate, *n* (%)	8 (28.5)	4 (13.8)	0.171

Quantitative data are presented as means ± standard deviations or numbers (percentages). ICU: intensive care unit.

## Data Availability

The datasets generated and/or analyzed during the present study are available from the corresponding author on reasonable request.
